# Exploring the impact of wheelchair design on user function in a rural South African setting

**DOI:** 10.4102/ajod.v4i1.171

**Published:** 2015-06-26

**Authors:** Surona Visagie, Svenje Duffield, Mariaan Unger

**Affiliations:** 1 Centre for Rehabilitation studies, Stellenbosch University, South Africa; 2 Division of Physiotherapy, Stellenbosch University, South Africa

## Abstract

**Background:**

Wheelchairs provide mobility that can enhance function and community integration. Function in a wheelchair is influenced by wheelchair design.

**Objectives:**

To explore the impact of wheelchair design on user function and the variables that guided wheelchair prescription in the study setting.

**Method:**

A mixed-method, descriptive design using convenience sampling was implemented. Quantitative data were collected from 30 wheelchair users using the functioning every day with a Wheelchair Scale and a Wheelchair Specification Checklist. Qualitative data were collected from ten therapists who prescribed wheelchairs to these users, through interviews. The Kruskal-Wallis test was used to identify relationships, and content analysis was undertaken to identify emerging themes in qualitative data.

**Results:**

Wheelchairs with urban designs were issued to 25 (83%) participants. Wheelchair size, fit, support and functional features created challenges concerning transport, operating the wheelchair, performing personal tasks, and indoor and outdoor mobility. Users using wheelchairs designed for use in semi-rural environments achieved significantly better scores regarding the appropriateness of the prescribed wheelchair than those using wheelchairs designed for urban use (*p* = <0.01). Therapists prescribed the basic, four-wheel folding frame design most often because of a lack of funding, lack of assessment, lack of skills and user choice.

**Conclusion:**

Issuing urban type wheelchairs to users living in rural settings might have a negative effect on users’ functional outcomes. Comprehensive assessments, further training and research, on long term cost and quality of life implications, regarding provision of a suitable wheelchair versus a cheaper less suitable option is recommended.

## Introduction

A wheelchair is defined by the WHO (2008:11) as ‘a device providing wheeled mobility and seating support for a person with difficulty in walking or moving about’. Thus, the purpose of a wheelchair is to improve personal mobility. With enhanced mobility comes the opportunity for greater function, access to services, community integration and employment (WHO 2008). However, function and community access is influenced by variables like the user's functional abilities, the environment and wheelchair design (Scherer 2002; Routhier *et al.* 2003; Vegter *et al.* 2010; Øderud 2014).

Part of the focus of this article is on the impact of wheelchair design on user function. Wheelchair design features, such as the overall length, weight, frame type and width, seat configuration, wheel and castor type, arm and footrests, axle position and propulsion mechanism, influence function (Vegter *et al.* 2010). There are five different categories of wheelchairs available on the South African National wheelchair tender, for prescription to users dependent on government health care services, (South African National Treasury 2010). The different design features of the five categories of wheelchairs and their impact on function are summarised in Table 1.

**TABLE 1:** Features of the different categories of wheelchair available on national tender.

Variables	Cruiser^®^	Pacer^®^	Econorigid^®^	Roughrider^®^	All-Terrain Wheelchair (ATW^®^) and World Made 3 (WM3^®^)
Frame type	Four-wheel folding frame	Four-wheel folding frame	Four-wheel rigid frame with fold-down backrest	Four-wheel folding frame	Three wheel rigid frame with fold-down backrest
Overall length (Using basic folding frame as standard reference)	Standard	Standard	Short	Short	ATW**^®^** - Standard WM3**^®^** - Long
Recommended use	Temporary use, and attendant propelled indoor and level outdoor terrain	Indoor and level outdoor terrain	Indoor and level outdoor terrain	Indoor, level and uneven outdoor terrain	Indoor, level and uneven outdoor terrain WM3**^®^** can handle rougher terrain
Indoor function (Using basic folding frame as standard reference)	Standard turn circle Difficult to manoeuvre in tight spaces	Standard turn circle Difficult to manoeuvre in tight spaces	Small turn circle. Compact and manoeuvrable in tight spaces	Smaller turning circle than Cruiser**^®^**	Larger turning circle. May limit indoor manoeuvrability, but narrow low boom fits under furniture and in tight spaces
Stability for outdoor use	Fixed high centre of mass and loading of front castors results in instability on uneven terrain.	Adjustable centre of mass User traverses uneven terrain by riding on rear wheels.	Low, adjustable centre of mass that is distributed over rear wheels User traverses uneven terrain by riding on rear wheels.	Low, adjustable centre of mass that is distributed over rear wheels Little load on front castors Users do not have to ride on rear wheels to travers uneven terrain.	As for Roughrider**^®^** Most stable on uneven terrain resulting from the three wheel design.
Propulsion ergonomics	No adjustability to enhance ergonomics	Can adjust seat vertically and horizontally	Can adjust seat vertically and horizontally	Can adjust rear wheel position horizontally	**ATW^®^** Adjust rear wheel position vertically and horizontally **WM3^®^** Adjust rear wheel position horizontally
Postural support	Though tension adjustable backrest	Through tension adjustable backrest, and adjusting rear axle settings	Through tension adjustable backrest, adjustable back height, front castor and rear axle settings	Through tension adjustable backrest and adjustable back height and rear axle settings	Though adjustable back height and angle
Transportability	Folds flat Footrests and armrests removable	Folds flat Footrests, armrests and rear wheels removable	Fold-down back rest, removable wheels, does not fold flat	Folds flat Similar to Cruiser with footplates and armrests removed	Fold-down back rest, removable wheels, does not fold flat Long boom requires more space
Cost		Cost on tender approximately R1000.00 (±$100) more than Cruiser**^®^**			

*Sources*: Provincial government of the Western Cape: Department of Health (PGWC DoH), 2009a, *Wheelchair Service Delivery Programme, Wheelchair Service Delivery Manual, Basic (professional) course,* PGWC DoH, Cape Town; Provincial government of the Western Cape: Department of Health (PGWC DoH), 2009b, *Product Manual*, PGWC DoH, Cape Town; Provincial Government of the Western Cape: Department of Health (PGWC, DoH), 2010, *Core package of care and standard operating procedures for wheelchair seating services,* PGWC DoH, Cape Town.

Design features must be matched to the user`s functional ability and posture support needs, and also to the environmental and durability requirements. Achieving an ideal match between user, wheelchair design and environment might be as difficult as it is important (Di Marco, Russel & Masters 2003).

Information from the wheelchair database of the Western region of the Eastern Cape Province (WREC) indicated that wheelchairs most suitable for indoor use and in flat outdoor environments (as are mostly found in urban areas) were mainly issued in this predominantly rural area. The reasons for this practice and its impact on user function are unknown. Thus, the objectives of the current study were to determine:

1. What the impact of wheelchair design was on user function and
2. What variables guided wheelchair prescription in this setting?

## Methodology

### Study design

A descriptive, mixed-method design was used (Kroll, Neri & Miller 2005). In the first phase of the study quantitative data were collected from wheelchair users to determine the type of wheelchair they received and their functional abilities in the wheelchair. In the second phase quantitative and qualitative data were collected, from physiotherapists and occupational therapists who prescribed these wheelchairs, to determine the factors that influence the type of wheelchair design they prescribe.

### Study setting

This study was performed in the WREC of South Africa. This region is similar to the rest of the Eastern Cape Province. Geographically it is a mountainous, hilly grassland environment, criss-crossed by rivers with muddy or sandy areas, depending on the season. The road infrastructure is poorly maintained and public transport is limited. Informal settlements are found throughout the region, with the majority of settlements in rural or semi-rural areas. Many people live in small ‘rondavel-type’ structures. Sanitary facilities and water is often shared and provided at strategic points in these settlements.

### Objective one: Impact of wheelchair design on user function

#### Study population and sampling

The 231 adults who lived in the WREC, and received a wheelchair from the Eastern Cape Department of Health (ECDoH) between 01 June 2010 and 30 June 2012, formed the study population. From this database 15 wheelchair users from rural areas and 15 from semi-rural areas were conveniently selected and invited to participate in the study. Users had to be 18 years or older for inclusion in the study, and needed to have had a government subsidised wheelchair for at least 3 months. Those with hired, loaned or privately financed wheelchairs were excluded.

#### Measuring instruments

The Functioning Everyday with a Wheelchair (FEW) scale (Mills, Holm & Schmeler 2007) and a wheelchair specification checklist (WSC) were used for data collection to address objective one. The FEW scale consists of three parts:

- Functioning Everyday with a Wheelchair (FEW/FMA questionnaire)
- Functioning Everyday with a Wheelchair-Capacity (FEW-C)
- Functioning Everyday with a Wheelchair-Performance (FEW-P).

The findings presented in this article focus on results from the FEW/FMA, which focuses on functional abilities and is completed by wheelchair users. It consists of 10 self-report items which are scored using a 6-point scale from 6 = completely agree to 1 = completely disagree.

The WSC consisted of two sections: Section A collates demographic data such as:

- diagnosis
- the period the user has been using the current wheelchair
- the occurrence of secondary complications like pressure ulcers.

Section B is a five category checklist to establish whether or not the prescribed wheelchair was appropriate. The categories are:

- size
- environment
- postural support
- function
- biomechanics.

The WSC was developed from the Provincial Government of the Western Cape's standards for wheelchair prescription (PGWC DoH 2009a). The checklist was peer-reviewed by a seating specialist^1^ to ensure content validity. Each category was scored on a 3-point scale. A score of ‘1’ meant the wheelchair was not suitable. A score of ‘2’ meant that the wheelchair was partially suitable or neutral to the needs of the user, and a score of ‘3’ meant the wheelchair was suitable.

#### Data collection

Participant's details were obtained from the WREC wheelchair database. Participants were contacted telephonically until 15 living in rural areas and 15 living in semi-rural areas consented to participate in the study. An appointment for data collection, at a venue of their choice, was made. On meeting the participants the study was explained to them, their questions were answered and written informed consent was obtained. Participants were asked to complete section A of the WSC and the FEW/FMA questionnaire. Thereafter section B of the WSC was administered. Questions were translated into isiXhosa by a translator where necessary.

## Data analysis

Quantitative data were analysed in consultation with a statistician from the Centre for Statistical Consultation (CSC) at Stellenbosch University (SU). Relationships between variables were tested with the Kruskal-Wallis test. A p-value of less than 0.05 was deemed statistically significant.

### Objective two: Variables that guided wheelchair prescription

#### Study population and sampling

The 14 therapists who issued wheelchairs to the users who participated in phase 1 of the study formed the study population for the second phase of the study. Two could not be identified as there was no signature on the requisition form. A further two were unreachable (one had emigrated and another did not return calls despite several attempts). The remaining ten therapists were contacted telephonically and all consented to participation.

#### Data collection tool

A self-compiled questionnaire, with open and close ended questions, was used to collect data from therapists. The questions focused on the therapists’ knowledge of wheelchairs available on tender and their perceptions of wheelchair prescription practice in the study setting. Of the ten therapists, nine had completed a basic wheelchair seating course and four had completed both a basic and an intermediate wheelchair seating course. These courses are based on the WHO guidelines for wheelchair provision in less resourced settings (WHO 2008; PGWC DoH 2009a, 2009b).

#### Data collection

Data were collected from the therapists through semi-structured interviews in English or Afrikaans, depending on the preference of the individual therapist. Interviews with therapists were electronically recorded and transcribed by an external scribe.

## Data analysis

Content analysis was used to identify emerging themes from the transcribed data. The different themes were highlighted in different colours, e.g. all text in the transcripts related to funding challenges was highlighted in green and coded as ‘Funding’ (Hsieh & Shannon 2005). A second rater identified themes independently and these were compared to the themes identified by the second author. Information from quantitative open-ended questions was summarised on a spreadsheet.

### Rigor

To add to the rigor of the data, triangulation of measuring instruments was done, e.g. function was determined by the FMA and WSC. All data were collected by one researcher. Generalisability of findings is negatively impacted by the small sample size, convenient sampling method and including only users with access to a telephone.

#### Ethical considerations

The study was registered with the Committee for Human Research at the Faculty of Health Sciences, Stellenbosch University (Ethics approval number: S12/08/231). In addition permission to perform the study was obtained from the Eastern Cape Department of Health and relevant institutional heads. Participation in the study was voluntary. Written informed consent was obtained from each participant. All information was treated as confidential.

## Results

### Impact of wheelchair design on user function

Of the 30 wheelchair users, six (20%) were female and 24 (80%) male. Their mean age was 43.4 years, with a minimum age of 19 and a maximum age of 82 years. The most common diagnosis was complete or incomplete spinal cord injury (47%), followed by lower limb amputation (23%).

As indicated in Table 2, 25 (83%) participants received a wheelchair more suitable for use in an urban environment and five (17%) received wheelchairs more suitable for use in ‘semi-rural’ environments.

**TABLE 2:** The type of wheelchair issued to participants (*n* = 30)

Urban wheelchair designs			Semi-rural wheelchair designs		Rural wheelchair design
Cruiser^®^	Adjustable four-wheel folding frame	Econorigid^®^	Roughrider^®^	All Terrain Wheelchair (ATW^®^)	World Made 3^®^ (WM3)
17 (57%)	0	8 (26%)	3 (10%)	2 (7%)	0

Table 3 shows that the size, fit, support and functional features of the wheelchair created transport challenges for 19 users. Thirteen (43%) wheelchair users had to hire private cars for transport. According to them taxi drivers (taxis in the setting are mainly minibus vehicles) refused to provide transport to wheelchair users, because it took much longer for a wheelchair user to transfer into the taxi and load their wheelchair than for an able-bodied client to embark. The loss of time has financial implications for the taxi owner. When asked about using a bus, users reported that they need help to embark and disembark as these vehicles are too high for independent transfers. Thus, someone had to accompany them.

**TABLE 3:** Ability to perform functional tasks in the wheelchair according to FMA scores.

Variable	Completely Agree (score 5–6)	Neutral (score 3–4)	Completely disagree (score 1–2)
Wheelchair features contribute to my ability to carry out daily routines	17	8	5
Size, fit, support and functional features of the wheelchair match my comfort needs	21	6	3
Size, fit, support and functional features of the wheelchair match my health needs	22	7	1
Size, fit, support and functional features of the wheelchair allows me to operate it independent, safely & efficiently	18	8	4
Size, fit, support and functional features of the wheelchair allows me to reach and carry out tasks at different surface heights	20	6	4
Size, fit, support and functional features of the wheelchair allows me to transfer from one surface to another	22	6	2
Size, fit, support and functional features of the wheelchair allows me to carry out personal care tasks	20	8	2
Size, fit, support and functional features of the wheelchair allows me to get around indoors	18	10	2
Size, fit, support and functional features of the wheelchair allows me to get around outdoors	18	7	5
Size, fit, support and functional features of the wheelchair allows me to use personal or public transport	11	13	6

Ten or more users experienced challenges in the categories of daily routine, operating the wheelchair, performing tasks at different surface heights, performing personal tasks, indoor mobility and outdoor mobility.

**FIGURE 1:**
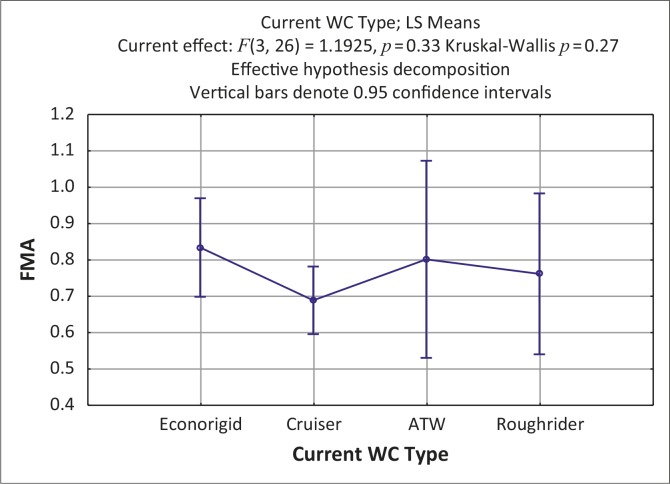
Impact of category of wheelchair on function (Kruskal-Wallis; *p* = 0.27)

As indicated in Figure 1, the type of wheelchair did not significantly impact FMA scores. The ATW^®^ had the widest range of scores with most users scoring low in the transport section and high in the outdoor mobility section. The Cruiser^®^ with basic, four-wheel, folding frame design had the lowest mean score and scored particularly low with regard to outdoor mobility, whilst the Econorigid^®^ (four-wheel, rigid frame design with adjustable settings and fold-down backrest) had the highest mean score.

**FIGURE 2:**
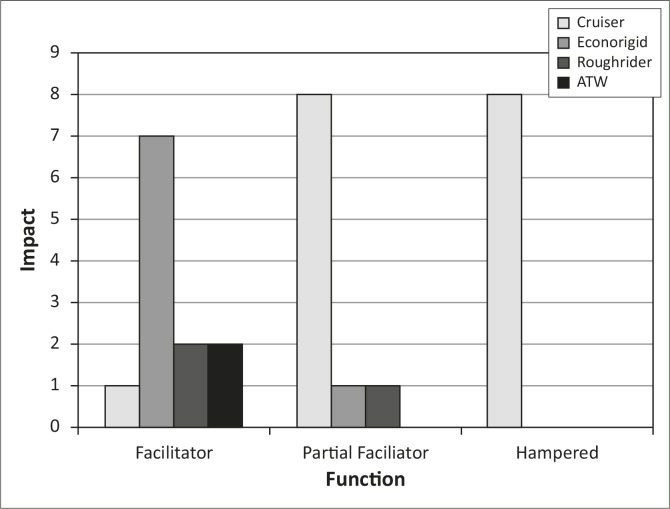
Impact of type of wheelchair on function as determined by the wheelchair specification checklist.

According to scores from the WSC five wheelchairs (all Cruisers^®^) were not suited to the environment of the user whilst eight (4x Cruisers^®^, 2x Econorigids^®^ and 2x Roughriders^®^) were suitable, and 17 suited the environment partially. Function was hampered for eight users all using Cruisers^®^, as shown in Figure 2, and facilitated for 12 users of whom seven used Econorigid^®^ wheelchairs.

**FIGURE 3:**
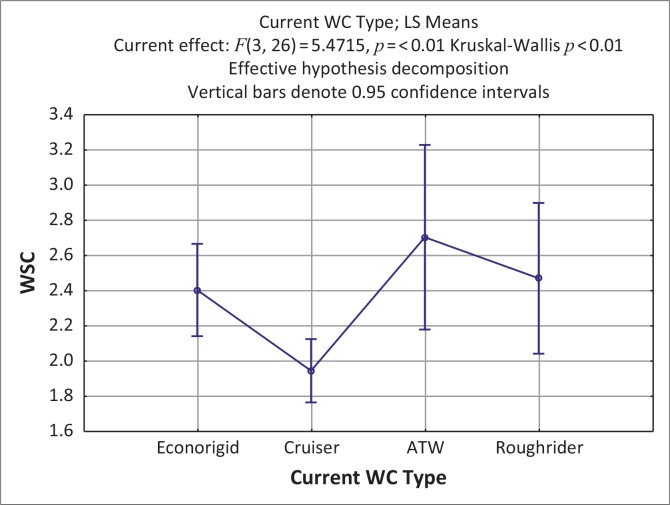
Comparison between wheelchair specification checklist scores and wheelchair design (Kruskal-Wallis; *p* = <0.01)

When assessing function during completion of the WSC it was found that more than half (57%) of the participants were unable to propel the wheelchair on even terrain, up and down an incline, or manoeuvre up and down a curb. Figure 3 shows that users using wheelchairs designed for use in semi-rural environments achieved significantly better WSC scores than users using wheelchairs designed for urban use (Kruskal-Wallis; *p* = <0.01).

### Variables that guided wheelchair prescription

All ten therapists who participated in the study indicated that they prescribed the basic four-wheel folding frame design (Cruiser^®^) most often. According to emerging themes this practice could mainly be ascribed to a lack of funding. Other factors that played a role included insufficient knowledge and skills, sub optimal assessments, inappropriate prescription, no design available on national tender that met all the needs of users, and user choice.

## Lack of funding

A lack of funding resulted in therapists prescribing cheaper designs, even if less appropriate than others, to increase their ability to provide more users with wheelchairs:

- ‘It's a moral dilemma – something is better than nothing, so you end up issuing what you have available instead of what is most appropriate.’ (Participant 8)
- ‘The restricted budget is a massive problem. A letter of concern was submitted through the region's Wheelchair Advisory Committee to the Rehabilitation Manager last year about this. No reply yet. We submit statistics on wheelchair orders every week to the CEO of our hospital, so that they are aware of the waiting list.’ (Participant 9)
- ‘I always think of the price before I order a wheelchair due to the budget constraints. I think before I order a specified wheelchair if it's not life changing, because those wheelchairs (wheelchairs with designs for semi-rural and rural use) are more difficult to recycle (re-issue to another user in the event of the first user passing away).’ (Participant 5)

Insufficient funding caused waiting periods in excess of 18 months:

- ‘Patients don’t get a wheelchair at the time of prescription, and two years later they probably need something completely different.’ (Participant 2)
- ‘By the time you receive the wheelchair and issue it the prescription isn’t accurate anymore because the patient and their circumstances have changed.’ (Participant 6)

## Inappropriate prescription

Lack of funding seems to cause inappropriate prescription that negatively impacted posture, function and wheelchair durability:

- ‘Poor funding for wheelchairs makes it impossible to issue the correct wheelchair at the appropriate time because there is such a long waiting list.’ (Participant 3)
- ‘In 2010 there was a gunshot wound patient who was put into a recliner wheelchair because it was all that was available. I saw how bad the wheelchair was for his posture and for his health. It was shocking.’ (Participant 2)
- ‘It's always so sad to see young or active clients going home in cruisers because it's the only wheelchair that is available at that time, sometimes it's not even the right size!’ (Participant 10)
- ‘Seeing Cruisers (basic four-wheel folding frame wheelchair) being returned or brought in for repairs and realising that they are not good enough for the harsh environments clients live in.’ (Participant 1)
- ‘Wrong prescriptions by other therapists and then I had to issue the wheelchair, and I knew the patient was going to be stuck with that wheelchair. I couldn’t just order them something more appropriate because the budget doesn’t allow that.’ (Participant 8)

### 

#### Insufficient skills

Some of the participants considered that lack of training and, therefore, a lack of appropriate skills amongst prescribing therapists caused problems to prescribing the most appropriate wheelchair design:

- ‘Yes, Cruisers (basic four-wheel folding frame wheelchair) are being ordered too often. It's a habit we have gotten into because we don’t know other wheelchairs, especially the newly qualified staff – their experience start with Cruisers and then they get stuck.’ (Participant 4)
- ‘Not enough product training from suppliers.’ (Participant 10)

#### Sub optimal assessment

Therapists reported, upon doing a home visit after issuing a wheelchair, that the wheelchair they had prescribed was completely inappropriate for the recipient:

- ‘I did a home visit and saw that the 20’ wheelchair couldn’t get into the bathroom or fit through the doorframe.’ (Participant 5)
- ‘(I) issued a wheelchair to a tetraplegic patient, and when I did a home visit (I saw) the patient couldn’t move around inside his house with this big wheelchair because the house was too small.’ (Participant 7)

Two (7%) of the users reported having had a home visit from a therapist or other medical professional. Therapists ascribed the lack of home visits to a shortage of transport and staff shortages.

#### Wheelchairs available on tender do not meet all user needs

Therapists felt that the wheelchairs currently available on tender do not necessarily meet all the needs of the wheelchair users:

- ‘…[*they are*] restricted to one wheelchair per client and one wheelchair can’t be appropriate to all areas of the client's life.’ (Participant 3)
- ‘If you issue a rural wheelchair to the patient their house is too small for it and transport is a big problem.’ (Participant 2)
- ‘The wheelchairs on tender are good, but environment where the patient lives makes it very difficult to select a wheelchair.’ (Participant 8)
- ‘The patient is very restricted in terms of transport – they want a folding wheelchair.’ (Participant 10)

#### User choice

- ‘[*I*] might feel ATW/other wheelchair is more appropriate but the client or the family want the Cruiser … Had a T12 spinal cord injury patient who was in a Cruiser and refused any other wheelchair.’ (Participant 8)
- ‘Access is a big problem and therefore patients often don’t want anything but a standard cruiser.’ (Participant 8)

## Discussion

Whilst the majority of users perceived themselves to be capable of performing all functional activities in the wheelchair, categories pertaining to daily activities, wheelchair dexterity and mobility created challenges for a third or more users. As the purpose for providing a wheelchair is to enhance function and mobility this finding remains worrying. The reasons for this can be multiple and might include a lack of training and a lack of physical ability (Vegter *et al.* 2010). Borg *et al.* (2012) found that training significantly decreased activity limitations and participation restrictions of wheelchair users,. However, functional challenges might also be related to wheelchair design, fit and biomechanical set up (Vegter *et al.* 2010; Medola *et al.* 2014; Øderud 2014).

Users using a basic four-wheel folding frame design experienced poorer overall function than those using other wheelchair designs. This may be because this design is not suitable for active users nor for outdoor use on uneven terrain. In addition this design provides little scope for biomechanical adjustments that could enhance user function (PGWC DoH 2009b; Medola *et al.* 2014). However, this design was the one most often issued and the wheelchair of choice for both therapists and users. This finding might be attributable to one or a combination of several factors. The basic four-wheel folding frame design was the only wheelchair available on tender, until 2000, and is better known to users and providers. Therapists predominantly attributed issuing this type of wheelchair ‘out of habit’. Some users considered this design was culturally and aesthetically more acceptable. In addition it is a small, foldable design that takes up less space inside buildings and is easier to transport (Medola **et al.* 2014)*. Finally, it was the cheapest option and funding challenges made therapists select it. Whilst appropriate in some instances, for example for the three users who were older than 60 and who had suffered a cerebro-vascular accident (their diagnosis and age are associated with lower activity levels (Steffen, Hacker & Mollinger 2002), it might have limited the function of more active users.

The four-wheel design with adjustable settings and fold-down backrest, which was issued the second most often, is considered appropriate for active wheelchair users in urban settings. The adjustable wheelbase of this design can assist with reducing the weight carried by the front castors and, thus, increase manoeuvrability of the wheelchair. In addition, optimal access to the rear wheel and, thus, more effective propulsion can be achieved through the adjustable settings. It is the experience of the authors that its greater manoeuvrability, lighter weight and transportability make this the wheelchair design of choice for many young, active users such as younger persons with spinal cord injuries (Dryden *et al.* 2003). However, the thin rear wheels and front castors, the low position of the footplate in relation to the ground and the short wheelbase make this design unsuitable to some rural and semi-rural environments (PGWC DoH 2009a). Despite this, many users living in a semi-rural environment were satisfied with this device, resulting from the wheelchair's lighter weight and centre of mass (COM) settings that enabled users to be highly active. Some users were using their wheelchairs for sport such as wheelchair basketball. Mason *et al.* (2010) found that professional wheelchair sportspeople considered stability to be the most important contributing factor towards performance, and this is a feature that the Econorigid^®^ wheelchair offers (PGWC DoH 2009b).

The ATW^®^ may be the more appropriate wheelchair for users living in a rural setting despite its potential access limitations in small houses. This will, however, need to be explored further as only two ATW^®^s were used by participants in this study. It is disquieting that none of the participants living in a rural setting were issued a World Made 3^®^ that was specifically designed for rural use. The overall size of the WM3^®^ and the difficulty of transporting it (PGWC DoH 2009b) might have influenced therapists and users to be less inclined to select this design than others.

Transport created a big challenge for user participants in the study. Other South African studies have reported similar findings (Chakwizira *et al.* 2010; Kahonde, Mlenzana & Rhoda 2010; Ntamo, Buso & Longo-Mbenza 2013). However, this might be attributable to factors other than wheelchair design. As described by current users, minibus taxi operators (the main source of public transport in the study setting) often refuse transport to wheelchair users or charge extra, because it takes longer for the person to transfer into the taxi and the wheelchair takes the room another paying passenger could have occupied.

The majority of users in this study, living in rural and semi-rural settings, received wheelchairs designed for urban use. Therapists reported that wheelchairs designed for urban use were issued most often in this setting. The findings of this study suggest that this design was not always inappropriate to the users’ environments and supports Vegter *et al.* (2010), as one cannot summarily equate a wheelchair design with an urban or rural setting and no single design can be seen as most optimal for a specific setting, as rural settings are not homogenous (Lourenço 2012).

A comprehensive assessment is required to determine appropriate design and should include a thorough investigation of the environments in which the user functions. This most likely will require a home, community and or work assessment visit (PGWC DoH 2009a). It seems from the findings as if a comprehensive assessment was not always performed. This omission may be why some users received wheelchairs not suitable to the environment in which they lived. Visagie, Scheffler & Schneider (2013) described assessment challenges which may negatively impact wheelchair prescription and overall wheelchair service delivery in a different South African setting. Asking the user about the home environment cannot replace an objective assessment. If one never used a wheelchair before you might not realise what aspects of the environment might create barriers and what wheelchair design options can best overcome these barriers. Therefore, subjective assessments of the user's home environment should not replace a home visit, especially in the case of first time users.

The results show that 17 participants were using a wheelchair that suited at least one aspect of their environment. Thus, a trade-off was needed in some circumstances; mostly between the need for a compact and manoeuvrable design in small indoor spaces and for transport, but a sturdy, stable design for rough outdoor surfaces. Therapists realised that the wheelchairs currently available on tender were not able to address all the needs of some users. This sentiment is supported by findings from Øderud (2014) in Zimbabwe. However, it might be that more training and practical experience is required to show therapists and users that, whilst bulkier, the low boom of the three wheel design offers advantages in indoor spaces, as presented in Table 1.

Amos & Winter (2013) argue that there is currently no wheelchair design that enables a user to travel both long distances over rough terrain and function in small indoor spaces. The therapists indicated two wheelchairs: one for indoor use and one for outdoor use that might be more appropriate in some circumstances. The findings of the current study, thus, support the rationale that many wheelchair users should have two wheelchairs (PGWC DoH 2009b). However, a lack of funding often prevented users from timeously accessing a wheelchair or from receiving the most optimal wheelchair design. Thus, issuing one user with two wheelchairs seems impossible. A similarly unsatisfactory and unacceptable solution would be to provide some users with more expensive wheelchairs whilst others receive nothing. Every user in need of a wheelchair should receive an appropriate wheelchair, even if the appropriate wheelchair is more expensive than the cheapest model that is available, and budgeting should be implemented accordingly.

Rural and semi-rural devices are more expensive than the basic, four-wheel, folding frame design and ordering these devices will deplete the wheelchair budget faster. Therapists indicated that they issued cheaper designs to ensure that more users are assured of receiving a wheelchair. Whilst this argument might seem reasonable, exhaustion caused by trying to propel a wheelchair, designed for urban use, over rugged terrain with narrow, steep footpaths and roads, might cause users to discard the wheelchair even if it is their only means of mobility (McAdam & Casteleijn 2005; Chakwizira *et al.* 2010). The issue of durability must also be considered. A wheelchair unsuitable to rugged terrain might break and need repairs and replacement more often, as described by one of the therapists; negating the initial saving of money (McAdam & Casteleijn 2005).

### Limitations

The non-parametric sampling procedure compromised the external validity of the study and generalisability of findings. Sample size was dictated by time and cost implications rather than power analysis. Thirty participants are too few to allow for extensive sub-group analysis in order to explore relationships between variables.

### Recommendations

More comprehensive assessments, including home and work visits, are recommended to allow for more appropriate selection of wheelchairs. In addition users functioned in two very distinct environments which require a wheelchair that is stable and functional on uneven terrain, yet manoeuvrable and compact in small dwellings. Further training of therapists and users is recommended, about the designs offered by the wheelchairs currently on tender and research into wheelchair design for promoting independent mobility in rural settings. Research is also recommended that looks into the long term cost and quality of life implications of providing a suitable wheelchair, versus providing a cheaper option that is less suited than others to the environment.

## Conclusion

The provision of wheelchairs, more suitable for urban use, to users living in rural settings might have impacted the functional outcomes of users adversely, especially in instances where the standard folding four-wheel design was prescribed. Reasons for prescribing the basic four-wheel folding frame wheelchair were being predominantly pragmatic, driven by cost, extended time-to-issue and fair distribution. User preference and different environmental needs experienced by the same user created challenges which the current system might be unable to address.

## Footnotes

1 BScOT; Manager CE Mobility EC.
